# Clinical Profile of Ingested Foreign Bodies in Children: A Single-Center Study

**DOI:** 10.7759/cureus.114955

**Published:** 2026-08-21

**Authors:** Kuriakose Shaji Paul, Sajith Sebastian, Jenny Susan Roy, Vijosh V Kumar, Ivy Abraham Varghese, Annup Balan Balathandan

**Affiliations:** 1 Medicine, Blackpool Victoria Hospital, Blackpool, GBR; 2 Department of Gastroenterology and Hepatology, Apollo Adlux Hospital, Kochi, IND; 3 Department of Periodontics and Implantology, Apollo Adlux Hospital, Kochi, IND; 4 Department of Gastroenterology, Aster MIMS, Kannur, IND; 5 Department of Gastroenterology, Sree Gokulam Medical College and Research Foundation, Venjarammoodu, IND; 6 Department of Infectious Diseases, Amrita Institute of Medical Sciences, Kochi, IND

**Keywords:** child safety, coins, cricopharyngeal region, endoscopic procedures, esophageal foreign bodies, ingestion of foreign bodies, paediatrics

## Abstract

Objective

This study aimed to evaluate the clinical characteristics, types, and locations of foreign bodies, as well as the clinical manifestations and outcomes following endoscopic extraction in pediatric patients.

Methodology

This prospective observational study included 75 children aged 0-12 years who presented with suspected foreign body ingestion at the Medical Trust Hospital, Kochi, Kerala, India. Detailed clinical histories, physical examinations, radiographic assessments, and endoscopic findings were recorded and analyzed.

Results

This study included 75 patients, including 35 (46.7%) males and 40 (53.3%) females. A statistically significant association was observed between gender and the site of impaction (p = 0.012). Lower esophageal involvement was noted in seven (20.0%) males and seven (17.5%) females. Foreign bodies were detected and endoscopically managed in 50 (66.7%) patients. A sensation of a foreign body was reported in 21 (84.0%) females and 12 (48.0%) males (p = 0.043). Coins were the most common objects, predominantly lodged in the post-cricoid region and the esophagus, whereas food impactions occurred exclusively in the esophagus, and needles were found in the stomach and duodenum. Endoscopic removal was successful in all cases within 24 hours, with no major complications. Five (6.7%) patients required a hospital stay of three days because of minor mucosal ulceration, which resolved with conservative management.

Conclusions

Foreign body ingestion is common among toddlers due to exploratory behavior and inadequate supervision. Coins and fish bones are the most frequently ingested items, with impaction sites and clinical presentations varying significantly by gender. Early diagnosis and prompt endoscopic removal are crucial for preventing complications. Public awareness and parental education play a vital role in reducing the incidence of these cases.

## Introduction

Foreign body ingestion and food bolus impaction are common presentations in clinical practice. Children younger than four years of age account for nearly 75% of reported cases [1]. In pediatric patients, ingestion is usually accidental and is brought to medical attention by their caregivers [2]. Commonly ingested objects include coins, button batteries, toys, magnets, safety pins, screws, marbles, and food boluses. Coins are the most frequently ingested foreign bodies worldwide [3].

Foreign body ingestion is relatively uncommon in adults but is frequently linked to specific risk factors [4]. These include advanced age, psychiatric disorders, alcohol intoxication, incarceration, and intentional ingestion for drug trafficking [5]. Approximately 80-90% of ingested foreign bodies pass spontaneously without requiring intervention. Endoscopic removal becomes necessary in 10-20% of cases, while surgery is required in fewer than 1% [6]. The indication for endoscopy depends on the patient’s clinical presentation and the type of foreign body ingested [7]. Although many patients are asymptomatic, those who develop symptoms typically present with manifestations related to the size, shape, and location of the object within the gastrointestinal tract [8]. Asymptomatic patients who ingest small, blunt objects can generally be managed with observation alone, except in cases involving batteries or magnets [9].

Imaging has a pivotal role in the evaluation of suspected foreign body ingestion. Plain radiography remains the initial diagnostic modality of choice, as it facilitates assessment of the size, number, and location of radiopaque objects. When radiographs are inconclusive, additional imaging modalities, such as ultrasonography or CT, may be utilized [10,11]. A recent study from Saudi Arabia found that among 117 pediatric patients, 92.9% underwent radiographic evaluation alone. Contrast swallow studies were performed in 3.5% of cases, and CT was required in 0.9% [12].

In India, data on pediatric foreign body ingestion are still scarce and often fragmented. Most available studies are limited to single-center experiences or small cohorts [13]. This limits robust international comparisons and hampers the development of evidence-based prevention strategies. In addition, many studies do not provide standardized reporting or sufficiently detailed clinical and procedural data. These gaps underscore the need for comprehensive institutional research to better characterize ingestion patterns and inform prevention strategies at both regional and national levels. The present study aims to address this gap by providing a comprehensive evaluation of pediatric foreign body ingestion at a tertiary care center in Kochi. It describes the patterns of presentation, types and sites of impaction, and outcomes of endoscopic management. Through this analysis, the study contributes to the expanding body of Indian literature on this important pediatric health issue.

## Materials and methods

Study design and setting

This prospective observational study was conducted at Medical Trust Hospital, Kochi, Kerala, India, over nine months from October 2018 to June 2019. All children who were evaluated and managed endoscopically for foreign body ingestion during this period were included in the study.

Eligibility criteria

ingestion involving the aerodigestive tract and who underwent either diagnostic or therapeutic endoscopy were considered eligible. Only patients with complete clinical records and properly documented parental consent were included. Children with foreign bodies located in the nose or ear, those with known congenital or acquired gastrointestinal abnormalities, patients requiring emergency surgical intervention without prior endoscopic evaluation, and cases with incomplete documentation or absence of consent were excluded.

Data collection procedure

A detailed history was obtained from the caregivers of all enrolled children, including age, sex, time elapsed since ingestion, suspected type of foreign body, and presenting complaints. Clinical symptoms assessed included foreign body sensation, dysphagia, odynophagia, vomiting, pooling of saliva, hematemesis, abdominal pain, and absence of symptoms. A thorough physical examination was performed in all patients. Radiological investigations, primarily plain radiographs of the neck, chest, and abdomen, were obtained when clinically indicated to identify the presence, number, and anatomical location of radiopaque foreign bodies. Demographic characteristics, clinical presentation, radiological findings, type of foreign body, anatomical site of impaction, endoscopic findings, retrieval device used, and treatment outcomes were recorded using a standardized data collection form.

Endoscopic evaluation and management

All patients underwent endoscopic evaluation under standard institutional anesthetic and monitoring protocols. The choice between rigid and flexible endoscopy was guided by the anatomical location, suspected nature of the foreign body, and the clinician’s discretion. Endoscopy was performed for both diagnostic confirmation and therapeutic retrieval of the foreign body. The anatomical site of impaction was recorded as the base of the tongue, pyriform fossa, post-cricoid region, upper esophagus, lower esophagus, stomach, first part of the duodenum (D1), or second part of the duodenum (D2).

Foreign bodies encountered included coins, fish bones, button batteries, metal pins, bottle caps, food boluses, needles, and other metallic objects. Retrieval devices, such as foreign body forceps, rat-tooth forceps, snares, Dormia baskets, and tripod baskets, were chosen based on the size, shape, and anatomical location of the foreign body. Successful retrieval, need for repeat procedures, duration of hospitalization, and procedure-related complications were documented.

Outcome measures

The primary outcome measures included the type and anatomical location of foreign bodies, clinical presentation, success of endoscopic retrieval, length of hospital stay, and procedure-related complications. Successful retrieval was defined as complete endoscopic removal of the foreign body without resorting to surgical intervention. Complications assessed included mucosal injury, ulceration, perforation, bleeding, and the need for additional interventions or procedures.

Data entry and statistical analysis

Data were entered into Microsoft Excel 2016 and subsequently analyzed. Categorical variables including age group, sex, clinical presentation, type of foreign body, site of impaction, and retrieval devices were summarized using frequencies and percentages. Associations between categorical variables were assessed using Fisher’s exact test because of the relatively small sample size and the presence of expected cell frequencies below five. Fisher’s exact test was used to evaluate the association between gender and site of foreign body impaction and between gender and clinical presentation among patients with confirmed foreign bodies. Because some patients presented with more than one symptom, symptom categories were analyzed as non-mutually exclusive variables, and frequencies could exceed the total number of patients. All statistical tests were two-tailed, and a p-value less than 0.05 was considered statistically significant.

## Results

This study included a total of 75 patients, comprising 35 (46.7%) males and 40 (53.3%) females, with a slight female predominance observed. In males, foreign bodies were most frequently located in the lower esophagus (n = 7, 20.0%) and the post-cricoid region (n = 7, 20.0%). In females, the lower esophagus and stomach were the most common sites, each seen in seven (17.5%) patients. Fisher’s exact test indicated a statistically significant association between gender and the site of foreign body impaction (p = 0.012). This finding highlights distinct distribution patterns, such as foreign bodies in the base of the tongue and D2 occurring exclusively in males, while stomach and D1 foreign bodies were observed only in females (Table 1).

**Table 1:** Gender distribution and site of foreign body impaction ^*^Indicates statistical significance Statistical significance was assessed using Fisher’s exact test. The values presented represent two-tailed p-values. A p-value < 0.05 was considered statistically significant

Site of occlusion	Male (n = 35), n (%)	Female (n = 40), n (%)	Total (n = 75), n (%)	P-value
Base of tongue	5 (14.3%)	0 (0%)	5 (6.7%)	0.012^*^
Pyriform fossa	2 (5.7%)	5 (12.5%)	7 (9.3%)	
Post cricoid area	7 (20.0%)	2 (5.0%)	9 (12.0%)	
Upper esophagus	2 (5.7%)	2 (5.0%)	4 (5.3%)	
Lower esophagus	7 (20.0%)	7 (17.5%)	14 (18.7%)	
Stomach	0 (0%)	7 (17.5%)	7 (9.3%)	
First part of the duodenum (D1)	0 (0%)	2 (5.0%)	2 (2.7%)	
Second part of the duodenum (D2)	2 (5.7%)	0 (0%)	2 (2.7%)	
No foreign body	10 (28.6%)	15 (37.5%)	25 (33.3%)	

A foreign body was detected and endoscopically managed in 50 (66.7%) of the 75 patients. Among males, odynophagia, foreign body sensation, and pooling of saliva were the most common presentations, each observed in 12 (48.0%) patients. In females, foreign body sensation was the most frequently reported symptom, noted in 21 (84.0%) cases. A small proportion of patients - two (8.0%) males and one (4.0%) female- presented with no complaints despite having a confirmed foreign body, underscoring the importance of a thorough history from caregivers even in asymptomatic children. Fisher’s exact test indicated a statistically significant association between gender and the clinical presentation of symptoms (p = 0.043) (Table 2).

**Table 2:** Clinical presentation of signs and symptoms in patients with confirmed foreign bodies ^*^Indicates statistical significance Some patients presented with multiple symptoms, so the total count exceeds the number of patients. Statistical significance was assessed using Fisher’s exact test. The values presented represent two-tailed p-values. A p-value < 0.05 was considered statistically significant

Sign of presentation	Male (n = 25), n (%)	Female (n = 25), n (%)	Total (n = 50), n (%)	P-value
Hememesis	0 (0%)	2 (8.0%)	2 (4.0%)	0.043^*^
Dysphagia	2 (8.0%)	5 (20.0%)	7 (14.0%)	
Vomiting	5 (20.0%)	2 (8.0%)	7 (14.0%)	
Odynophagia	12 (48.0%)	7 (28.0%)	19 (38.0%)	
Pooling of saliva	12 (48.0%)	5 (20.0%)	17 (34.0%)	
Abdominal pain	5 (20.0%)	2 (8.0%)	7 (14.0%)	
Foreign body sensation	12 (48.0%)	21 (84.0%)	33 (66.0%)	
No complaints	2 (8.0%)	1 (4.0%)	3 (6.0%)	

The relationship between the type of foreign body and its anatomical site of impaction in these 50 patients revealed distinct patterns. Coins were the most frequently ingested object, with the highest concentration in the post-cricoid region and esophagus. Fish bones were primarily found in the upper aerodigestive tract, specifically at the base of the tongue and pyriform fossa. Food impactions occurred exclusively in the esophagus. Needles, demonstrating their potential for distal migration, were found in the stomach and duodenum (Table 3).

**Table 3:** Types of foreign bodies and their sites of impaction in patients with confirmed foreign bodies (n = 50) ^*^The total number of foreign bodies (58) exceeds the number of patients (50) because some participants might have ingested more than one type of foreign body

Type of foreign body	Base of tongue	Pyriform area	Post-cricoid area	Esophagus	Stomach	Duodenum	Total
Fish bone	9	2	0	0	0	0	11
Coin	0	3	5	4	3	0	15
Bottle cap	0	0	2	0	0	0	2
Battery	0	0	0	3	2	0	5
Food impaction	0	0	0	10	0	0	10
Polyp	0	0	0	3	0	0	3
Needles	0	0	0	0	4	5	9
Metal piece	0	0	0	3	0	0	3
Total	9	5	7	23	9	5	58^*^

A total of 58 endoscopic retrieval procedures were performed. Foreign body forceps were used in 21 procedures, with a retrieval success rate of 94%. Dormia baskets were used in 18 procedures, also achieving a 94% success rate. Rat-tooth forceps were used in 10 procedures, with a 90% success rate, while snares were used in eight cases, yielding an 88% success rate. Although the tripod basket demonstrated a lower apparent success rate, it was used in only one procedure; therefore, no reliable conclusions regarding its comparative effectiveness could be made. Overall, foreign body forceps and Dormia baskets demonstrated the highest retrieval success rates (Table 4).

**Table 4:** Type of devices used for foreign body retrieval in patients with confirmed foreign bodies (n = 50) ^*^The total number of procedures (58) exceeds the number of patients (50) because some participants required the use of more than one type of device to achieve the successful retrieval of the foreign body

Device used	No. of procedures	Success rate
Rat tooth forceps	10	90%
Foreign body forceps	21	94%
Snare	8	88%
Dormia basket	18	94%
Tripod basket	1	50%
Total	58^*^	

All patients underwent successful endoscopic evaluation, and foreign body removal was completed within 24 hours of admission. The procedure was highly effective, with no major complications, such as perforation or a need for surgical intervention. The majority of patients were discharged within 24 hours of the procedure. A small subset of patients (n = 5, 6.7%) required a prolonged hospital stay of three days for observation and management of minor mucosal ulcerations at the site of the previous impaction, all of which resolved without further intervention.

## Discussion

Foreign body ingestion is a common pediatric emergency that can lead to serious complications if not promptly identified and managed [1]. Young children are particularly at risk due to their exploratory behavior and tendency to place objects in their mouths. Early recognition and timely intervention are essential to prevent mucosal injury, airway obstruction, and other complications [3]. Despite the extensive literature on this topic, ingestion patterns and outcomes vary by region and population, highlighting the importance of local observational studies.

In our single-center study of 75 children with suspected foreign body ingestion, 40 (53.3%) were female, and 35 (46.7%) were male, demonstrating a slight female predominance in this study cohort. Given the single-center design and limited sample size, this finding should not be interpreted as evidence of a true population-level gender predominance. Ingestion occurred most frequently in children aged two to four years. A foreign body was detected and endoscopically managed in 50 (66.7%) patients. Coins were the most commonly ingested objects, accounting for 28.0% of cases with a foreign body. Most patients presented with symptoms, primarily a foreign body sensation (66.0%) and odynophagia (38.0%), while 34.0% had pooling of saliva.

A statistically significant association was observed between gender and both the site of impaction (p = 0.017) and clinical presentation (p = 0.046). Endoscopic retrieval was successful in more than 90% of procedures using various devices. Mucosal injury was not systematically assessed, and no button battery ingestions were recorded in this cohort. The selection of retrieval instruments depended on the size, shape, and anatomical location of the foreign body. However, subgroup comparisons of device performance for identical foreign body types were limited by the relatively small and heterogeneous sample.

Comparing our findings with the literature, Gupta et al. [14] reported that 91% of upper aerodigestive foreign bodies occurred in children under 10 years of age. Coins were the predominant esophageal foreign body, and foreign body sensation was the most common symptom. Saliva pooling was observed in a similar proportion of patients. Both studies demonstrated low complication rates and high success rates with endoscopic removal. Our findings that coins were the most frequent foreign body and that foreign body sensation was the predominant symptom align closely with their observations.

Ding G et al. [15] reported a male predominance among patients with aspirated foreign bodies in the larynx, trachea, and bronchi, whereas our cohort showed a slight female predominance. Children under three years of age were at the highest risk in both studies. When comparing symptom profiles, Ding et al. [15] reported cough, dyspnea, and wheezing, whereas foreign body sensation and odynophagia predominated in our cohort. This difference is expected, given that their study focused on aspirated foreign bodies requiring bronchoscopy, whereas ours focused on ingested foreign bodies managed with upper gastrointestinal endoscopy.

Coins and fish bones were the most common foreign bodies in our study, whereas Ding et al. [15] reported frequent aspirations of peanuts and other organic material. The differences between the two studies may be attributed to variations in study population characteristics, regional epidemiology, referral patterns, and the inclusion of different categories of foreign body presentations. Variations in gender predominance across studies may reflect regional demographic differences, referral patterns, and sampling variability rather than a true biological predisposition.

Bajaj D et al. [16] described cases of foreign body aspiration requiring ICU care and bronchoscopic procedures. Similar to our findings, rapid identification and removal were critical for preventing complications. Both studies successfully used instruments such as forceps and baskets. Our cases did not require advanced tools such as cryoprobes or lasers. Post-procedural evaluation remained important for confirming complete removal and identifying mucosal injury, which was observed as minor ulcerations in 6.7% of our patients, all of which resolved without intervention.

Martha et al. [17] reported that most foreign body ingestions occurred in children under five years of age. While our study observed a slight female predominance, their cohort showed a male predominance. The observed gender distribution may also reflect local population demographics and referral patterns. Therefore, the slight female predominance identified in this cohort should be interpreted cautiously. Vomiting and gastrointestinal pain were more common in their study, whereas foreign body sensation and odynophagia predominated in ours. Some patients presented solely with a history of ingestion without immediate symptoms, highlighting the importance of thorough evaluation even in asymptomatic children. Both studies reported favorable outcomes with high recovery rates and no fatalities.

Button battery ingestion is increasingly recognized as a high-risk event, particularly when the battery is lodged in the nose, esophagus, stomach, or external ear canal. Although only five cases (10.0% of detected foreign bodies) were recorded in our cohort, previous studies [18,19] have reported serious complications, including nasal septal and tympanic membrane perforations. Gatto et al. [20] reported high success rates with endoscopic removal, emphasizing the importance of early diagnosis and timely intervention. Our experience with button battery removal was similarly successful, with no complications, likely due to prompt presentation and intervention within 24 hours.

The high endoscopic removal success rate in our study (over 90% across all device types) is consistent with the published literature [6,7]. Foreign body forceps were the most commonly used device (34.5% of procedures), with a 94% success rate, followed by the Dormia basket (30.9% of procedures), which also achieved a 94% success rate. The choice of device depended on the type, size, and location of the foreign body, emphasizing the importance of maintaining a range of retrieval instruments for effective management.

Strength and limitations 

A major strength of this study is its prospective design and comprehensive evaluation of the clinical presentation, anatomical distribution, endoscopic management, and outcomes of foreign body ingestion in a pediatric population managed at a tertiary care center. However, several limitations should be acknowledged. First, the study was conducted at a single center and included a relatively small sample size, which may limit the generalizability of the findings. Second, long-term follow-up was not performed, which precluded the assessment of delayed complications and long-term outcomes after foreign body ingestion and removal. Third, symptom documentation relied partly on caregiver-reported histories and clinical records, which may have introduced reporting bias. Additionally, some patients presented with multiple symptoms, resulting in overlap among symptom categories and potentially affecting the interpretation of symptom-based analyses.

The observed gender distribution should also be interpreted cautiously, as it may have been influenced by local population demographics and referral patterns rather than reflecting a true biological predisposition. Similarly, the spectrum of foreign bodies encountered was likely influenced by local environmental, cultural, dietary, and behavioral factors, which may limit direct comparison with studies from other geographic regions. Furthermore, age-related exploratory behavior may have influenced the pattern of foreign body ingestion and clinical presentation. However, age-stratified analyses were limited by the sample size. Finally, although Fisher’s exact test was selected because of the small sample size and sparse categorical data, the observational nature of the study limits causal inferences regarding associations between demographic and clinical variables.

## Conclusions

This study describes the clinical profile, anatomical distribution, and outcomes of pediatric foreign body ingestion in a tertiary care setting. Foreign body ingestion occurred most commonly in young children, with coins being the most frequently encountered objects. Early endoscopic evaluation and removal resulted in a high success rate and a low incidence of complications. While statistically significant associations were observed between gender and certain clinical characteristics, these findings should be interpreted cautiously given the study’s sample size, single-center design, and the potential influence of local demographic factors. Larger multicenter studies with long-term follow-up are needed to further characterize risk factors, evaluate management strategies, and improve preventive interventions for pediatric foreign body ingestion.
